# Effects of Berberine Plus Inulin on Diabetes Care in Patients With Latent Autoimmune Diabetes in Adults: Protocol for a Randomized Controlled Trial

**DOI:** 10.3389/fendo.2022.876657

**Published:** 2022-06-15

**Authors:** Rong Zhang, Yang Xiao, Jianru Yan, Wen Yang, Xiaomei Wu, Zubing Mei, Zhiguang Zhou

**Affiliations:** ^1^ National Clinical Research Center for Metabolic Diseases, Key Laboratory of Diabetes Immunology, Ministry of Education, and Department of Metabolism and Endocrinology, The Second Xiangya Hospital of Central South University, Changsha, China; ^2^ Department of Endocrinology, The First People’s Hospital of Pingjiang, Pingjiang, China; ^3^ Department of Anorectal Surgery, Shuguang Hospital Affiliated to Shanghai University of Traditional Chinese Medicine, Shanghai, China; ^4^ Anorectal Disease Institute of Shuguang Hospital, Shanghai, China

**Keywords:** berberine, inulin, glycemic control, diabetes care, LADA (latent autoimmune diabetes in adults), randomized controlled trial

## Abstract

**Background:**

Latent autoimmune diabetes in adults (LADA) is a heterogeneous form of diabetes, characterized by autoimmune destruction of pancreatic β-cells as well as insulin resistance and is triggered by environmental factors in the context of genetic susceptibility. Berberine (BBR), a small alkaloid isolated from medicinal plants, has antidiabetic, anti-inflammatory, and antibacterial effects. Inulin is a common prebiotic that has been shown to improve glycemic control, alter the gut microbiota and suppress inflammation. The primary purpose of this study was to evaluate the effects of oral BBR and inulin combined with insulin therapy on diabetes care in patients with LADA.

**Methods and Analysis:**

We will conduct a single-center, prospective, randomized, double-blind, placebo-controlled trial. A total of 240 patients with LADA who have started insulin therapy will be randomly allocated either to the intervention or control group. After a 1-week run-in period, they will complete a 3-month treatment of BBR alone, inulin plus BBR, inulin alone, or placebo. Anthropometric and clinical data will be collected at five time points: baseline, 3 months, 6 months, 9 months, and 12 months from baseline. The primary outcome was the change in glycated hemoglobin levels. Dynamic blood glucose parameters, β-cell function, and gut microbiota, as well as adverse events and quality of life will be monitored.

**Discussion:**

Glycemic control is critical for preventing the progression of diabetes. Although insulin is a recommended treatment for patients with LADA, there are currently no drugs that can effectively prevent the progressive destruction of pancreatic β-cells or maintain their function. Several studies have found that when berberine and prebiotics are used alone, they have beneficial metabolic effects. This clinical research protocol will assess the efficacy of the combined treatment of berberine plus inulin and provide new ideas for future pharmacological research and clinical practices in diabetes care and glycemic control for LADA patients.

**Ethics and Dissemination:**

This study has been approved by the Ethics Committee of National Clinical Research Center of the Second Xiangya Hospital of Central South University (approval number: 2021–046).

**Clinical Trial Registration:**

ClinicalTrials.gov, identifier NCT04698330

## 1 Introduction

Due to the increased aging population and prolonged life expectancy, the global prevalence of chronic diseases and their complications has caused a huge burden on the socioeconomic and medical costs. Latent autoimmune diabetes in adults (LADA) is a chronic disease that shares the common phenotypic, genetic and pathophysiological features of type 1 diabetes (T1D) and type 2 diabetes (T2D) (1). LADA is typically diagnosed using three criteria proposed by the Immunology of Diabetes Society: (a) adult age of onset (>30 years); (b) the presence of any islet cell autoantibodies, and (c) insulin independence for at least 6 months following diagnosis (2). Diabetes prevalence has risen dramatically in recent years worldwide, and LADA may account for 2–12% of all diabetes in adults (3). Epidemiological studies have shown that there are approximately 6 million patients with LADA in China (4), and LADA patients account for one-tenth of the total population with diabetes in some regions, such as Northern Europe (5). Therefore, the drug treatment and blood glucose management of LADA patients are worthy of our attention.

LADA is characterized by the destruction of pancreatic β-cells caused by autoimmunity and insulin resistance (6). Due to differences in genetics and immunity, LADA has a high degree of heterogeneity. When compared to classic T1D, the autoimmune process of LADA appears to be milder and patients with LADA progress more slowly toward β-cell failure and insulin therapy (7, 8). As a result, health education for LADA patients is essential to promoting good blood glucose management and preserving their remaining β-cell function. Good glycemic control is crucial to prevent future complications and improve patients’ quality of life.

However, a portion of LADA cases can often be misdiagnosed as T2D, with misdiagnosis rates ranging from 5% to 10% in various studies (9). Moreover, misdiagnosed LADA cases are usually treated with therapies commonly used in T2D, which might contribute to the progression of diabetes toward insulin dependence and threaten the health and life of patients (10). Since LADA individuals generally have some residual β-cell function, LADA treatment should aim to achieve good metabolic control while also retaining residual insulin secretion capacity (11). Currently, insulin is the most commonly used therapeutic option for LADA patients. Several randomized clinical trials have found that regardless of the initial C-peptide level and residual β-cell function, clinicians tend to prescribe insulin treatment for LADA patients at an early stage (12). However, whether insulin is truly appropriate as the initial treatment for LADA patients is still debatable. Although LADA patients have better metabolic parameters at baseline than T1D and T2D patients (13), evidence suggests that there is almost no difference in the prognosis of complications and risk of death among the three groups (14–16). Given that insulin alone may not be sufficient to control blood glycemic levels, combination therapy may have the potential to improve long-term blood glucose control and delay disease progression in LADA patients. Because there have been few clinical studies on LADA, more large-scale clinical trials are needed to explore the optimal treatment of LADA.

BBR, an alkaloid compound extracted from many commonly used medicinal plants, such as Coptis chinensis and Berberis asiatica, has been shown in some basic and clinical research to play an important role in alleviating metabolic disorders, improving insulin resistance, regulating intestinal flora, inhibiting inflammation and attenuating intestinal mucosal barrier dysfunction (17–20). A recent study by Jun Yin et al. enrolled 36 adults with new-onset T2D and randomly assigned them to either the BBR or metformin treatment groups (21). They found that similar to the hypoglycemic effect of metformin, BBR can significantly reduce hemoglobin A1c (HbA1c), fasting blood glucose (FBG), postprandial blood glucose (PBG) and plasma triglycerides at the end of the three-month treatment period. The same group of researchers conducted another study in which 48 T2D patients with poor glycemic control were treated with berberine, and they found that the supplementary treatment reduced the subjects’ HbA1c and improved their insulin resistance (21). Furthermore, BBR has low gastrointestinal absorption and a high safety profile, with few adverse reactions and the ability to be tolerated by the human body (22). All of these findings demonstrate BBR’s efficacy as a new candidate drug in the treatment of diabetes and other metabolic diseases.

Inulin, a fructooligosaccharide mixture, is a common prebiotic that has been made into a food grade brewed drink powder. Many studies have found that prebiotics have beneficial metabolic effects in diabetes. A randomized controlled clinical trial in Iran in which 52 women with T2D were randomly assigned to receive oligofructose-enriched inulin or placebo for 8 weeks found that oligofructose-enriched inulin improved the patients’ glycemic control and inflammatory status (23). Josephine Ho et al. conducted a clinical study among children aged 8 to 17 with T1D who received placebo or oligofructose-enriched inulin for 12 weeks (24) and found that C-peptide levels were significantly higher in the prebiotic treatment group, which was accompanied by a moderate improvement in intestinal permeability.

In animal experiments, it has been shown that compared with BBR alone, BBR combined with prebiotics has been shown to continuously reduce FBG, improve glucose tolerance, and ameliorate the balance of α- and β-cells in diabetic db/db mice (25). Similarly, Cao et al. found that the combination of BBR and prebiotics in the intervention of KKAy mice was more effective than BBR alone in blood sugar control, improvement of insulin resistance and pancreatic β-cell function, reduction of inflammatory mediators, and maintenance of intestinal barrier integrity (26). These findings suggest that the combination of BBR and prebiotics may be a novel pharmaceutical strategy for diabetes. Hence, the present study aims to evaluate the effectiveness of combining BBR and inulin on diabetes care, particularly glycemic control in patients with LADA. We will also evaluate the safety and tolerability of the combined drugs.

## 2 Methods

### 2.1 Trial Design

**Figure 1:**
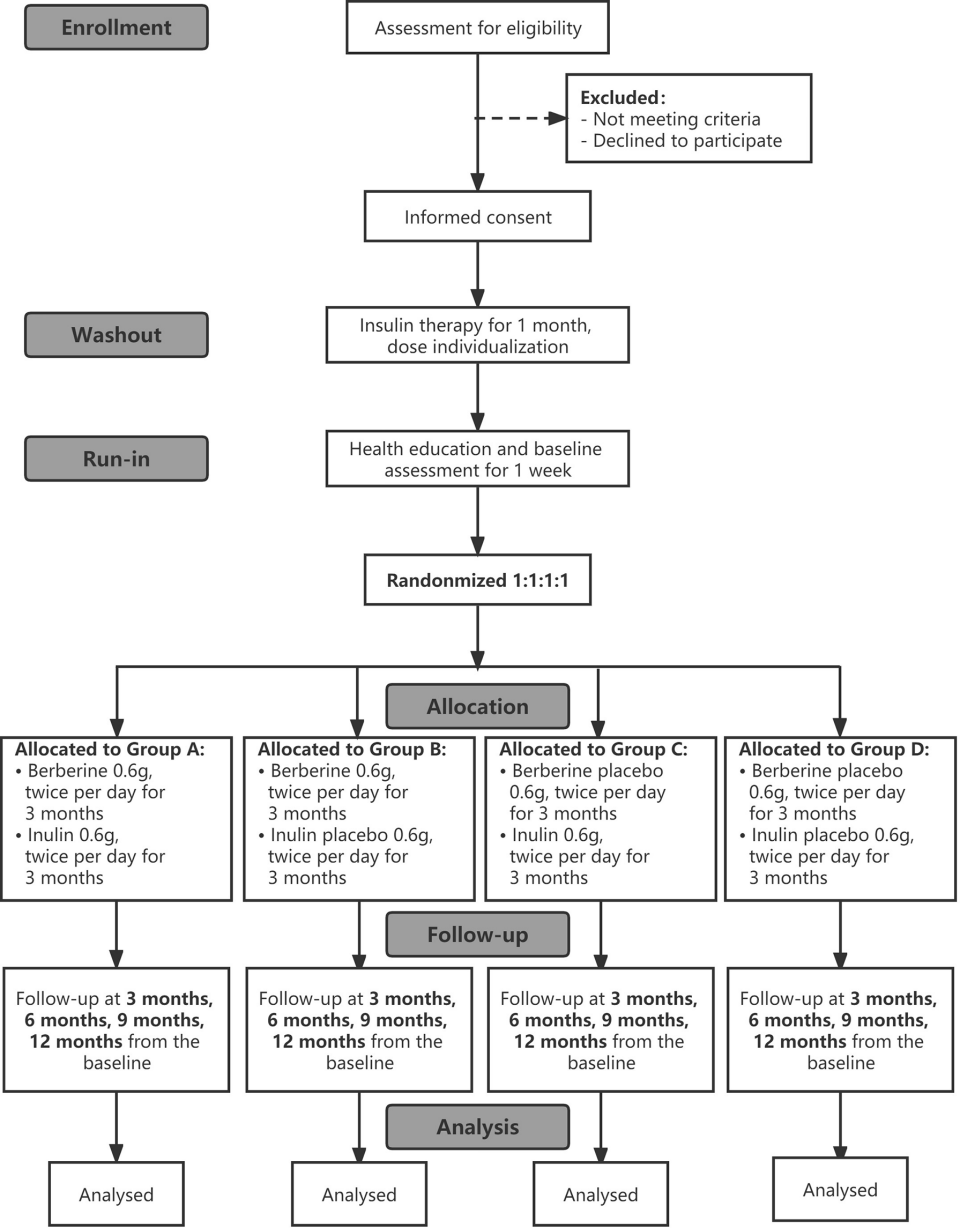
Trial flowchart.

This is a prospective, randomized, double-blinded, placebo-controlled trial with a 1-month washout period, a 1-week run-in period, a 3-month treatment period, and a 9-month follow-up period. The participants will be arranged to enter a 1-month washout period after obtaining their informed consent during which basic treatment medications are adjusted, and then they will be allocated to enter a 1-week run-in period for lifestyle education and collection of baseline data and samples. After the run-in period, they will be randomized 1:1:1:1 to one of four groups: BBR-alone, inulin plus BBR, inulin-alone, or placebo. This study will use a stratified random method to balance the following factors: BMI ≥25 kg/m^2^ and <25 kg/m^2^, with or without chronic complications of diabetes. Measurements will be implemented at baseline prior to the intervention and at 3 months, 6 months, 9 months and 12 months from baseline. The research flowchart is shown in **Figure 1**.

#### 2.1.1 Participants

The target population of the trial will be LADA patients between the ages of 30 and 70. Participants will be recruited at a large tertiary first-class hospital in Hunan, China. The following inclusion criteria will be used in the recruitment:

Participants of both sexes aged 30 to 70 with a BMI between 18.5 and 37.5 kg/m^2^; with diabetes diagnosed according to the diagnostic criteria of the World Health Organization (WHO) in 1999; positive glutamic acid decarboxylase antibody (GADA) and HbA1c level ≥ 7.0% and ≤ 10.0%; being insulin-independent for more than 6 months after diagnosis; diabetes duration less than 5 years; fasting serum C-peptide (FCP) level ≥0.15 nmol/L, or 2-hour postprandial C-peptide (2hCP) level ≥0.3 nmol/L; and providing written informed consent will be included.

Participants will be excluded from the trial if they (1) have severe liver dysfunction (ALT and AST levels greater than three times the upper limit of detection); (2) have an eGFR < 50 ml/min/1.73 m^2^; (3) have evidence of acute or chronic infection affecting glycemic control within 4 weeks prior to the first visit; (4) have a history of any malignancy; (5) are pregnant/breastfeeding women; (6) have secondary diabetes; (7) have acute complications (ketoacidosis, lactic acidosis or hyperosmolar coma); (8) have severe organic heart disease, including but not limited to congenital heart disease, rheumatic heart disease, hypertrophic or dilated cardiomyopathy, etc., New York Heart Association (NYHA) heart function classification ≥Grade III; (9) have used corticosteroids or immunosuppressants for over 3 months, or other medications that can cause gastrointestinal reactions 2 months before recruitment and throughout the whole study period; (10) have a history of hemolytic anemia or glucose-6-phosphate dehydrogenase deficiency; and (11) are allergic to berberine hydrochloride or any components in the combinations.

#### 2.1.2 Sample Size Determination

Sample size was determined using published data from a 12-week clinical trial with BBR and probiotics, in which the drugs decreased HbA1c by 0.44% in people with T2D (Zhang, Gu et al., 2020), assuming a common within-group standard deviation (SD) of 0.80% and a two-sided α level of 0.05. With these assumptions, a sample size of 53 evaluable subjects per arm provided 80% power to detect a difference from placebo. Considering the reasonable attrition during the study, we planned to enroll 60 patients per group (i.e., a total of 240 patients).

### 2.2 Randomization

Once the participants are enrolled, they will be allocated a unique identification number according to their screening order. Participants will be distributed into four treatment groups in a 1:1:1:1 ratio through computerized randomization. A web administrator will independently enter the randomization list, containing consecutive drug numbers, into a blinded datasheet. The numbers will be randomly allocated to participants of the intervention groups or the control group with an opaque, sealed envelope by a third person unaware of the assignments to study groups.

### 2.3 Interventions

All participants who sign the informed consent will first enter a 1-month washout period during which insulin is used as basal therapy to avoid interference of previous hypoglycemic therapy on the study results. The proportion of patients treated with insulin will be comparable in each group. According to the individual characteristics of each subject, the investigator and the physician in charge should determine the brand, dosage form, initial injection dose and frequency of insulin. The optimal glycemic control target is fasting blood glucose (FBG) in the morning <7.2 mmol/L and 2 hours postprandial blood glucose (PBG)<10.0 mmol/L. The dose and dosage form of insulin will be adjusted or switched based on the subjects’ average blood glucose level over the three days preceding each visit. Other antidiabetic drugs will be prohibited throughout the study. After completing the screening and evaluation, the research nurses will implant the dynamic blood glucose monitor subcutaneously for the participants and advise them on the appropriate nursing measures. Prior to the intervention, participants will receive a 1-week diabetes health education as well as a baseline assessment. Then, they will be randomly divided into the following four groups: BBR (0.6 g per 6 pills, twice daily before meals) plus inulin (0.6 g per 6 pills, twice daily before meals), BBR plus placebo (inulin), inulin plus placebo (BBR), or placebo (inulin) plus placebo (BBR). BBR is manufactured by Yabang Epson Pharmaceutical Co., Ltd., Yancheng, Jiangsu, China. Inulin was purchased from Sino Peptide Health Technology Co., Ltd., Beijing, China, and its raw material supplier was Cosucra in Belgium. If desired, inulin can be processed into different dosage forms. The placebos are similar in appearance to BBR and inulin. Each type of tablet is packaged uniformly in an opaque sealed bottle with instructions and labels. The care providers and participants will be blinded to the assignment of medicine. After 3 months of treatment, participants will be asked to return the drug packages to determine their compliance. Researchers will conduct regular follow-up by telephone with all the participants to encourage and improve medication compliance, and record related adverse events including hypoglycemia and gastrointestinal symptoms.

### 2.4 Outcome Assessment

#### 2.4.1 Primary and Secondary Outcomes

The primary outcome measure is the change in mean HbA1c level, measured at baseline and 3 months post-intervention, which reflects the blood glucose management status of the participants.

The secondary outcome measures include: (1) the frequency of hypoglycemic events and drug-related side effects; (2) the change in C-peptide for assessing β-cell function and the incidence of acute and chronic diabetes complications; (3) daily insulin dose and medical service utilization, such as diabetes-related hospital admissions and emergency visit rate; (4) the alteration of gut microbiota assessed by multiomics measurements and the differences in intestinal permeability; and (5) the quality of life assessed by the Audit of Diabetes Dependent Quality of Life (ADDQoL-19) containing physical functioning, symptoms, psychological well-being, social well-being, role activities and personal constructs with its Chinese version CN-ADDQoL also having the individualized properties, satisfactory reliability and validity (Cronbach’s alpha=0.941) (26).

#### 2.4.2 Demographics, Anthropometric and Clinical Data

Demographic information will be collected at baseline, including age, sex, nationality, occupation, marital status, residence and education level. Anthropometric measures mainly include body mass index (BMI), waist and hip circumference, heart rate and blood pressure. Clinical data including blood lipids, blood biochemical findings, immune and inflammation markers and imaging examinations will be gathered from baseline and specified visit points.

#### 2.4.3 Data Collection

After the screening, all qualified participants with LADA will enter a 1-month washout period. After the stable control of diabetes with insulin, all enrolled participants will complete the baseline survey for a period of 1 week. The primary endpoint results will be collected after 3 months of intervention. Then, participants will receive outpatient visits every 3 months until the end of the study. The research assistants involved in data collection and evaluation will not be informed of the grouping of participants. The evaluation and data collection process is shown in **Table 1**. The participants will record their daily dose of medications, abnormal self-monitoring of blood glucose levels, combined medications and adverse events. Before each visit, participants will be asked to fill out an online survey of nutritional status in the past week to assess calorie intake, including the content of carbohydrates, fat, protein and fiber in the diet. Nurses will instruct patients to record the type and quantity of daily food *via* online and mobile platforms. In addition, it is worth noting that participants should not consume fermented dairy products (such as yogurt) or probiotics 7 days before the visit and should avoid high-fat diets 3 days before the visit.

**Table 1:** Patient flow of enrolment, intervention and outcome measurements.

		PS	M0	M3	M6	M9	M12
**Enrolment**	Recruitment	×					
	Eligibility Screening	×					
	Informed consent	×					
	Allocation		×				
**Intervention**	Insulin injection	•					•
	Experimental drugs / placebo		•	•			
**Outcomes**	Demographic characteristics (Age, sex, nationality, occupation, marital status, residence and education level)		×				
	Anthropometric variables (BMI, waist and hip circumference, heart rate and blood pressure)	•					•
	Insulin logs		•				•
	Dietary logs		•				•
	CGMS*		•				•
	Metabolic parameters (HbA1C, FBG, PBG, FCP, 2hCP, Triglyceride, Total cholesterol, HDL cholesterol, LDL cholesterol)	•					•
	HOMA-IR**		•				•
	Insulin autoantibody (GADA, IA2A, ZnT8)	×	×	×			×
	Serum inflammation markers		×	×			
	Chronic complication screening (Carotid ultrasound, UCG, fundus examination, urinary albumin-creatinine ratio, eGFR)		×				×
	Gut microbiota (Metagenomics, stool metabolomics, plasma metabolomics)		×	×			×
	Intestinal permeability		×	×			×
	Drug safety assessment (Blood routine, Liver and kidney function testing, Serum electrolytes, Blood ketones, electrocardiogram)		×	×			
	Quality of Life Questionnaire***		×	×			×
	Adverse events record (hypoglycemic events and drug-related side effects)		×	×			

PS, prior to study; M0, baseline assessment; M3, 3 months from the baseline; M6, 6 months from the baseline; M9, 9 months from the baseline; M12, 12 months from the baseline. *CGMS, continuous glucose monitoring system; **HOMA-IR, homoeostasis model assessment index for assessing insulin resistance; ***questionnaire: CN-ADDQoL.

### 2.5 Data Analysis

Data analysis will be performed using SPSS 26.0 software (IBM, New York, USA). P<0.05 was regarded as statistically significant unless otherwise specified. Descriptive statistics included the mean ± standard deviation for normal distributions, median and interquartile range for otherwise, and frequency and percentage for categorical variables. Intention-to-treat analysis (ITT) will be used and multiple imputations will be handled to manage missing data. Baseline descriptive data between the control group and the intervention groups will be compared using the chi-square test for categorical variables, and ANOVA for continuous variables. The primary outcome of HbA1c will be expressed as the mean HbA1c values with standard deviations, and ANOVA will be used to compare HbA1c between groups. According to the data distribution characteristics, ANOVA or Wilcoxon rank sum test will be used to compare the changes in secondary variables within and between groups. If the difference is significant, pairwise comparisons will be performed with adjustment for multiple comparisons, and ANCOVA will be used with confounding factors such as sex and age as covariates. Spearman’s correlation analysis and partial Spearman’s correlation analysis adjusted for sex and age will be used to evaluate the relationship between changes in intestinal microbial abundance and other markers.

## 3 Discussion

Due to the rapid rate of population aging and advances in medical technology that increase life expectancy and reduce disease mortality, the management of chronic diseases and their associated complications is a difficult issue that requires strong support from the government and society. If the treatment is poor, it will greatly reduce the quality of life of the patients and cause long-term physical and mental damage. Multiple previous studies have assessed the long-term risk of macrovascular and microvascular complications in LADA patients. In the United Kingdom Prospective Diabetes Study (UKPDS), LADA patients were found to have a lower long-term risk of major adverse cardiovascular events than patients with new-onset T2D, mainly due to their younger mean age and more favorable cardiometabolic status (27). In addition, the research team also found that in the first 9 years after diabetes diagnosis, patients with LADA had a lower risk of microvascular complications (renal failure, renal death, blindness, vitreous hemorrhage, or retinal photocoagulation), but this situation was reversed by persistently poor glycemic control thereafter, with an adjusted risk of 33% higher in patients with LADA than in T2D patients (28). Similarly, Maddaloni et al. noted that LADA patients had a lower risk of developing cardiac autonomic neuropathy than T2D patients (29). These evidences highlight the existence of an early therapeutic window in LADA to improve complication outcomes by implementing strict glycemic control.

Qianjin huanglian pill is composed of Coptis chinensis powder and fresh Rehmanniae radix, which has been a classic prescription for treating diabetes since ancient times. The hypoglycemic effect of this traditional Chinese medicine may be partly attributed to one of its main components, BBR. Clinical studies have demonstrated that BBR not only exerts a hypoglycemic effect by regulating the structure and function of the human gut microbiota, but also improves fasting and postprandial lipidemia in T2D patients to achieve better cardiovascular risk control (30, 31). Animal experiments have also shown that BBR has important potential in the treatment of diabetes-related complications such as diabetic nephropathy (32, 33), diabetic retinopathy (34, 35), diabetic cardiomyopathy (36), diabetic encephalopathy (37) and diabetic neuropathy (38) by inhibiting inflammation and oxidative stress, anti-endothelial injury, regulating glucose and lipid metabolism, inhibiting microvascular proliferation and regulating autophagy. Inulin has been reported to suppress inflammation and oxidation, modulate glucose and lipid metabolism, regulate immunity, modulate gut microbiota and improve intestinal barrier function (39, 40). Studies have shown that prebiotics can enhance the beneficial effects of BBR on glucose metabolism by modulating intestinal microbiota and their metabolites (26). Moreover, the increased Langerhans cells (LCs) in the cornea and epidermis of LADA patients with peripheral neuropathy also suggest that inflammation plays an important role in the development of diabetes-related complications (41), and the anti-inflammatory properties of BBR and inulin make them advantageous in the synergistic treatment of LADA complications. Thus, the identification of compatible ingredients to delay the progression of diabetes from natural products with metabolic regulation potential will provide effective and brand-new means for the clinical treatment of LADA and its complications. However, there remains a lack of clinical evidence in this regard in the LADA population.

Undeniably, this study has several limitations. First, the trial will be conducted only in the LADA population in China. Therefore, the results may not be directly applied to people of other races and need to be further validated by other regional or international multicenter studies in the future. Second, due to the difficulties in recruiting LADA patients, it is also not easy to maintain the balance between groups, and some participants are LADA patients who have already started drug treatment, so the results of this study may not be applicable to new-onset and mild LADA patients. In addition, the study will last for approximately one year, and the specific time of insulin use in LADA patients should be judged based on the clinical experience of the clinicians. Finally, the long-term effect of the study drugs on the gut microbial condition and complication risk of the patients is also unclear.

In conclusion, the present study will provide clinical evidence to prove the treatment effectiveness and safety of supplementation with BBR plus inulin in LADA patients. In addition, if combination medication is shown to improve blood glucose management and patient quality of life, insights gained from this study will provide new clues for future pharmacological research and clinical practices in diabetes care and glycemic control.

## Data Availability Statement

The original contributions presented in the study are included in the article/supplementary material. Further inquiries can be directed to the corresponding authors.

## Ethics Statement

The studies involving human participants were reviewed and approved by the Ethical Review Committee of Second Xiangya Hospital of Central South University (approval number: 2021–046). The patients/participants provided their written informed consent to participate in this study.

## Author Contributions

YX, ZM, and RZ designed the study and drafted the manuscript. ZZ, JY, WY, and XW edited and revised the manuscript. ZM and YX reviewed the methodology and the whole content of the manuscript. All the authors have read and approved the submission of the final version of the manuscript which meets at least one of the following criteria recommended by the ICMJE* (http://www.icmje.org/recommendations/): (1) substantial contributions to conception and design, acquisition of data or analysis and interpretation of data; (2) drafting the article or revising it critically for important intellectual content.

## Funding

This work was supported by the National Key Research and Development Program of China (2018YFE0114500 to YX), the National Natural Science Foundation of China (81870577 to YX), the National Science Foundation of Hunan Province for Excellent Young Scholars (2020JJ3056 to YX), the science and technology innovation Program of Hunan Province (2021RC3032 to YX).

## Conflict of Interest

The authors declare that the research was conducted in the absence of any commercial or financial relationships that could be construed as a potential conflict of interest.

## Publisher’s Note

All claims expressed in this article are solely those of the authors and do not necessarily represent those of their affiliated organizations, or those of the publisher, the editors and the reviewers. Any product that may be evaluated in this article, or claim that may be made by its manufacturer, is not guaranteed or endorsed by the publisher.
